# Crystal structures and hydrogen bonding in the anhydrous tryptaminium salts of the isomeric (2,4-di­chloro­phen­oxy)acetic and (3,5-di­chloro­phen­oxy)acetic acids

**DOI:** 10.1107/S205698901500907X

**Published:** 2015-05-23

**Authors:** Graham Smith, Daniel E. Lynch

**Affiliations:** a Science and Engineering Faculty, Queensland University of Technology, GPO Box 2434, Brisbane, Queensland 4001, Australia; b Exilica Ltd., The Technocentre, Puma Way, Coventry CV1 2TT, England

**Keywords:** crystal structure, tryptamine salts, phen­oxy­acetic acids, herbicides, 2,4-D, 3,5-D, hydrogen bonding

## Abstract

The anhydrous salts of 2-(1*H*-indol-3-yl)ethanamine (tryptamine) with isomeric (2,4-di­chloro­phen­oxy)acetic acid (2,4-D) and (3,5-di­chloro­phen­oxy)acetic (3,5-D), both C_10_H_13_N_2_^+^·C_8_H_5_Cl_2_O_3_^−^ [(I) and (II), respectively], have been determined and their one-dimensional hydrogen-bonded polymeric structures are described. In the crystal of (I), the aminium H atoms are involved in three separate inter-species N—H⋯O hydrogen-bonding inter­actions, two with carboxyl­ate O-atom acceptors and the third in an asymmetric three-centre bidentate carboxyl­ate *O,O′* chelate [graph set *R*_1_^2^(4)]. The indole H atom forms an N—H⋯O_carboxyl­ate_ hydrogen bond, extending the chain structure along the *b*-axis direction. In (II), two of the three aminium H atoms are also involved in N—H⋯O_carboxyl­ate_ hydrogen bonds similar to (I) but with the third, a three-centre asymmetric inter­action with carboxyl­ate and phen­oxy O atoms is found [graph set *R*_1_^2^(5)]. The chain polymeric extension is also along *b*. There are no π–π ring inter­actions in either of the structures. The aminium side-chain conformations differ significantly between the two structures, reflecting the conformational ambivalence of the tryptaminium cation, as found also in the benzoate salts.

## Abstract 2

The anhydrous tryptaminium salts of isomeric (2,4-di­chloro­phen­oxy)acetic acid and (3,5-di­chloro­phen­oxy)acetic acid give one-dimensional hydrogen-bonded chain structures which differ both in their cation–anion conformations and modes of inter-ion inter­action.

## Chemical context   

2-(1*H*-Indol-3-yl)ethanamine (tryptamine) is an alkaloid found in plants and fungi and is a possible inter­mediate in the biosynthetic pathway to the plant hormone indole-3-acetic acid (Takahashi, 1986 ▸). It is also found in trace amounts in the mammalian brain, possibly acting as a neuromodulator or neurotransmitter (Jones, 1982 ▸). As a relatively strong base (p*K*_a_ = 10.2), it readily forms salts with a number of organic acids. To investigate the modes of hydrogen-bonding inter­action in crystals of the tryptaminium salts of ring-substituted phen­oxy­acetic acid analogues, the reaction of tryptamine with two isomeric homologues, the herbicidally active (2,4-di­chloro­phen­oxy)acetic acid (2,4-D) (Zumdahl, 2010 ▸) and (3,5-di­chloro­phen­oxy)acetic acid (3,5-D), gave the anhydrous salts, C_10_H_13_N_2_^+^·C_8_H_5_Cl_2_O_3_^−^, (I) and (II), respectively. Their structures and hydrogen-bonding modes are reported herein. The structure of the anhydrous salt with phen­oxy­acetic acid (Koshima *et al.*, 1999 ▸) represents the only reported example of a salt from this acid series. In that crystal, chirality was generated through hydrogen bonding, giving cation–anion units related along a 2_1_ screw axes. A similar phenomenon was also observed in the tryptaminium 4-chloro­benzoate crystal (Koshima *et al.*, 2005 ▸).
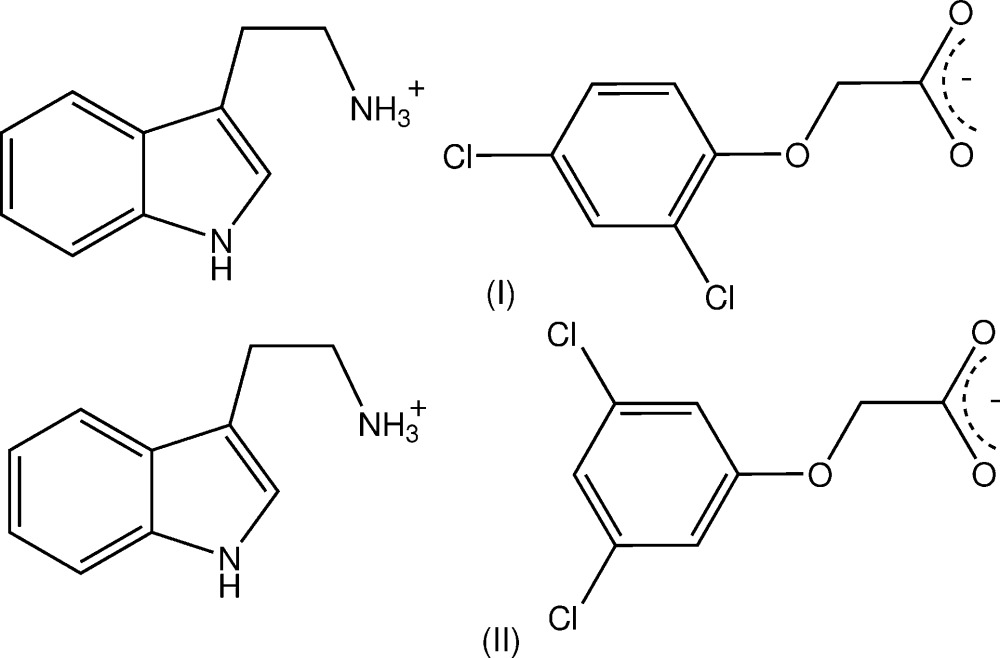


## Structural commentary   

**Figure 1:**
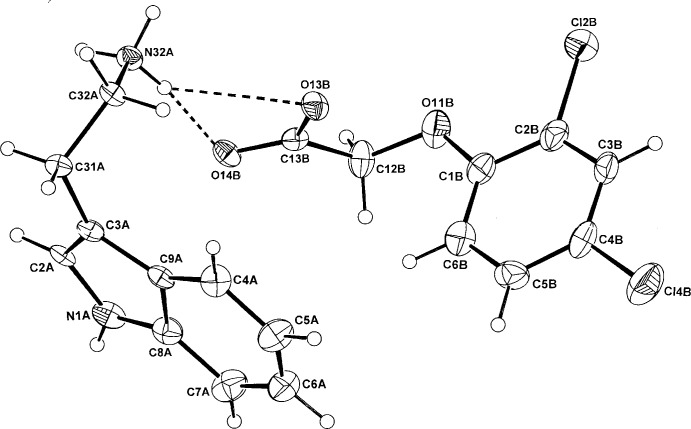
The atom-numbering scheme and the mol­ecular conformation of the TRYP^+^ cation (*A*) and the 2,4-D^−^ anion (*B*) in (I) with displacement ellipsoids drawn at the 40% probability level. The cation–anion hydrogen bonds are shown as dashed lines.

**Figure 2:**
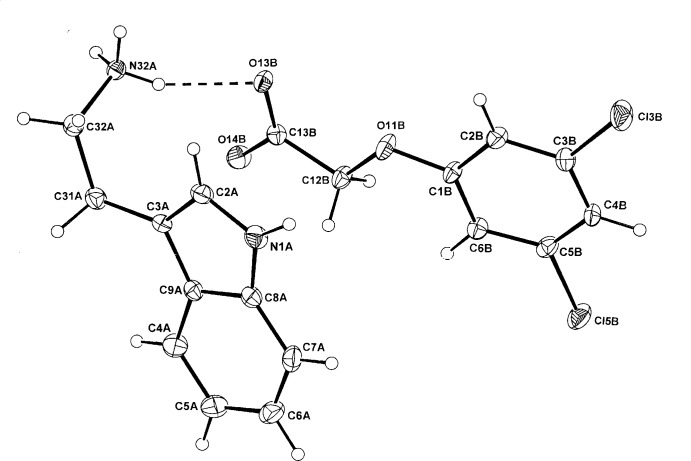
The atom-numbering scheme and the mol­ecular conformation of the TRYP^+^ cation (*A*) and the 3,5-D^−^ anion (*B*) in (II) with displacement ellipsoids drawn at the 40% probability level. The cation–anion hydrogen bond is shown as a dashed line.

The asymmetric units of (I) and (II) comprise a tryptaminium cation (*A*) and either a 2,4-di­chloro­phen­oxy­acetate anion (*B*) (I) (Fig. 1 ▸) or a (3,5-di­chloro­phen­oxy)acetate anion (II) (Fig. 2 ▸). Unlike a number of tryptaminium salts of benzoic acids in which the benzene rings in the cation and anion species are essentially parallel, giving π–π inter­actions, these planes in (I) and (II) are not so [dihedral angles = 74.1 (3) and 24.68 (17)°, respectively], giving no π–π inter­active effects.

The alkyl­aminium side chains in the cations of (I) and (II) differ significantly, with the torsion angles C2*A*—C3*A*—C31*A*—C32*A* and C3*A*—C31*A*—C32*A*—C32*A*—N32*A* being −113.1 (5), 58.6 (5)° in (I), 7.3 (5) and in 75.7 (4)° (II), respectively. This variability is a standard feature in the structures of the known tryptaminium benzoate salts, which include the parent benzoate (Terakita *et al.*, 2004 ▸), 4-chloro­benzoate (Koshima *et al.*, 2005 ▸), 3,4-di­meth­oxy­benzoate (Siripaisarnpipat & Larsen, 1987 ▸), 3,5-di­nitro-2-hy­droxy­benzoate (Lynch *et al.*, 2015 ▸) and the pseudopolymorphic anhydrous, mono- and dihydrate 3,5-di­nitro­benzoates salts (Lynch *et al.*, 2015 ▸). In the structure of tryptamine, determined from powder diffraction data (Nowell *et al.*, 2002 ▸), the corres­ponding angles are −89.4 (6) and 60.7 (6)°.

In (I) the phen­oxy­acetate side chain of the 2,4-D anion is significantly rotated out of the benzene plane [defining torsion angle C1*B*—O11*B*—C12*B*—C13*B* = 81.2 (6)°], similar to that of the parent acid which also has the *synclinal* side chain conformation (torsion angle 90±30°) (comparative torsion angle = 75.2°; Smith *et al.*, 1976 ▸). However, in the potassium salt (Kennard *et al.*, 1983 ▸) and the ammonium salt (Liu *et al.*, 2009 ▸) (both hemihydrates), the *anti­periplanar* (180±30°) conformation is found. The 3,5-D anion in (II) adopts the *anti­periplanar* conformation with the defining C1*B*—O1*B*—C12*B*—C13*B* torsion angle = −166.5 (3). The structure of the parent acid is not known but the equivalent angle in the ammonium salt is −171.35 (15)° (Smith, 2015 ▸) but in the 2:1 adduct of 3,5-D with 4,4′-bi­pyridine (Lynch *et al.*, 2003 ▸), the angle is −71.6 (3)° (*synclinal*).

## Supra­molecular features   

**Figure 3:**
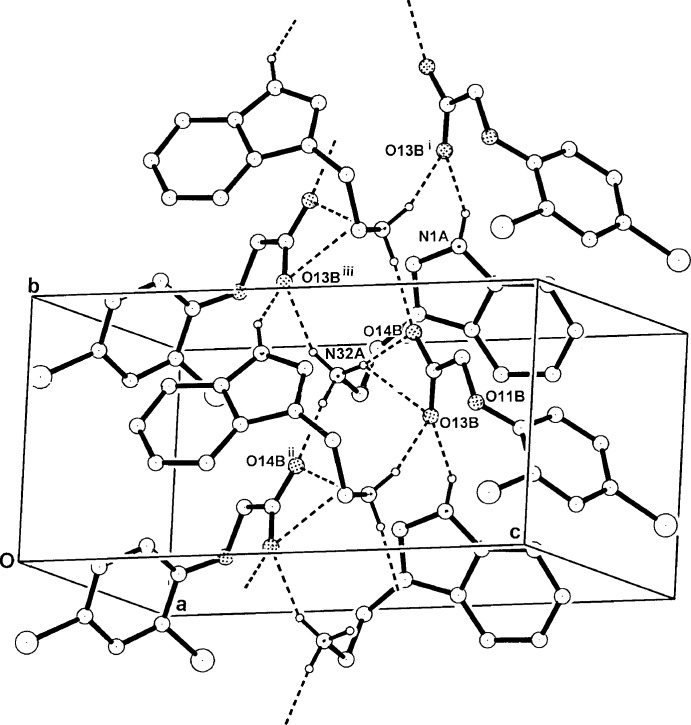
The one-dimensional hydrogen-bonded polymeric structure of (I) extending along [010], with non-associative H atoms omitted. For symmetry codes, see Table 1 ▸.

**Figure 4:**
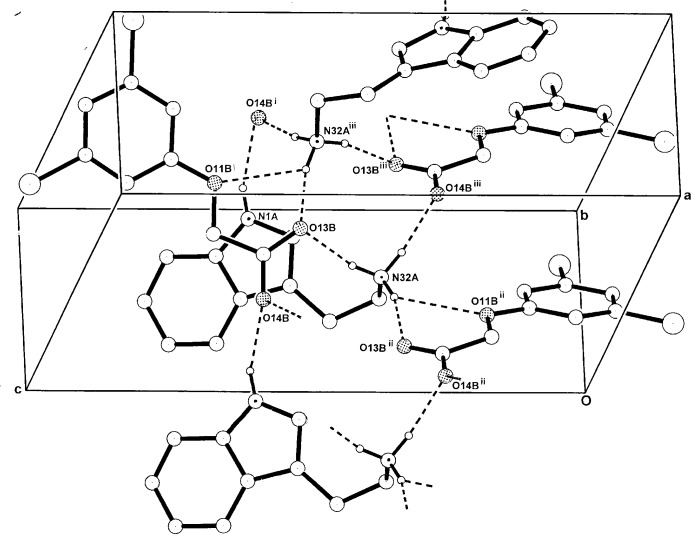
The one-dimensional hydrogen-bonded polymeric structure of (II) extending along [010], with non-associative H-atoms omitted. For symmetry codes, see Table 2 ▸.

In the crystal structures of (I) and (II), one-dimensional hydrogen-bonded structures involving N—H⋯O_carboxyl­ate_ inter­actions are found. However, the hydrogen-bonding patterns differ significantly. In the crystal of (I), the three aminium H atoms give different inter-species inter­actions, two with single carboxyl­ate O-atom acceptors (O13*B* ^iii^, O14*B* ^ii^) and third giving a three-centre *O,O′* chelate with carboxyl­ate O atoms (O13, O14) [graph set *R* ^2^_1_(4)] (Table 1 ▸). The indole H atom gives an N—H⋯O_carboxyl­ate_ hydrogen bond, extending the chain structure down the [010] axis (Fig. 3 ▸). In the crystal of (II), as with (I), two of the three aminium N—H⋯O inter­actions are with single carboxyl­ate O atoms [(O13*B*, O14*B* ^iii^) but the third differs in that it forms a three-centre asymmetric inter­action with carboxyl­ate and phen­oxy O atoms of the anion (O13*B* ^ii^, O11*B* ^ii^) [graph set 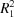
(4)] (Table 2 ▸). The chain polymeric N1—H⋯ O14*B* extension is also along [010] (Fig. 4 ▸).

The present pair of structures of salts of tryptamine with isomeric (2,4-di­chloro­phen­oxy)acetic acid and (3,5-di­chloro­phen­oxy)acetic acid provide examples which further reflect the conformational ambivalence of the cationic alkyl­aminium side chain of the tryptamine cation, shown also in the benzoate salts.

## Synthesis and crystallization   

The title compounds (I) and (II) were prepared by warming together for 2 min, solutions containing equimolar qu­anti­ties of (2,4-di­chloro­phen­oxy)acetic acid (2,4-D) or (3,5-di­chloro­phen­oxy)acetic acid (3,5-D) (138 mg) with 100 mg of tryptamine in ethanol. Room temperature evaporation of the solutions gave in both cases, colourless needles of (I) and (II) from which specimens were cleaved for the X-ray analyses.

## Refinement details   

Crystal data, data collection and structure refinement details are given in Table 3 ▸. Hydrogen atoms were placed in calculated positions [C—H_aromatic_ = 0.95 Å or C—H_methyl­ene_ = 0.99 Å] and were allowed to ride in the refinements, with *U*_iso_(H) = 1.2*U*_eq_(C). The aminium H atoms were located in difference-Fourier analyses and were allowed to refine with bond length restraints [*d*(N—H = 0.88 (2) Å], and with *U*_iso_(H) = 1.2*U*_eq_(N). Although possibly not of relevance in these crystals involving achiral mol­ecules, the Flack absolute structure factors (Flack, 1983 ▸) were determined as 0.01 (7) for (II) (2232 Friedel pairs) and 0.45 (15) for (I) (1619 Friedel pairs), in the case of (I) suggesting possible racemic twinning. No indication of conventional twinning was found with the crystals of either isomer.

## Supplementary Material

Crystal structure: contains datablock(s) global, I, II. DOI: 10.1107/S205698901500907X/sj5460sup1.cif

Structure factors: contains datablock(s) I. DOI: 10.1107/S205698901500907X/sj5460Isup2.hkl

Structure factors: contains datablock(s) II. DOI: 10.1107/S205698901500907X/sj5460IIsup3.hkl

Supporting information file. DOI: 10.1107/S205698901500907X/sj5460Isup4.cml

Supporting information file. DOI: 10.1107/S205698901500907X/sj5460IIsup5.cml

CCDC references: 1400285, 1400284

Additional supporting information:  crystallographic information; 3D view; checkCIF report

## Figures and Tables

**Table 1:** Hydrogen-bond geometry (Å, °) for (I)

*D*—H⋯*A*	*D*—H	H⋯*A*	*D*⋯*A*	*D*—H⋯*A*
N1*A*—H1*A*⋯O13*B* ^i^	0.87 (4)	2.13 (5)	2.879 (6)	144 (6)
N32*A*—H34*A*⋯O14*B* ^ii^	0.89 (4)	1.89 (4)	2.782 (6)	175 (2)
N32*A*—H35*A*⋯O13*B* ^iii^	0.90 (4)	2.10 (5)	2.817 (6)	137 (4)
N32*A*—H36*A*⋯O13*B*	0.89 (3)	2.57 (4)	3.231 (6)	132 (4)
N32*A*—H36*A*⋯O14*B*	0.89 (3)	1.94 (4)	2.816 (6)	171 (5)

Symmetry codes: (i) 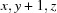; (ii) 
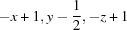
; (iii) 
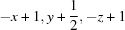
.

**Table 2:** Hydrogen-bond geometry (Å, °) for (II)

*D*—H⋯*A*	*D*—H	H⋯*A*	*D*⋯*A*	*D*—H⋯*A*
N1*A*—H1*A*⋯O14*B* ^i^	0.87 (4)	2.04 (4)	2.838 (4)	152 (4)
N32*A*—H34*A*⋯O13*B*	0.87 (2)	2.05 (3)	2.875 (4)	160 (4)
N32*A*—H35*A*⋯O11*B* ^ii^	0.89 (3)	2.60 (4)	3.160 (4)	122 (3)
N32*A*—H35*A*⋯O13*B* ^ii^	0.89 (3)	1.87 (3)	2.739 (4)	164 (4)
N32*A*—H36*A*⋯O14*B* ^iii^	0.89 (4)	1.90 (4)	2.775 (4)	170 (4)
C2*A*—H2*A*⋯O13*B* ^iii^	0.95	2.55	3.495 (4)	177

Symmetry codes: (i) 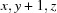; (ii) 
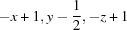
; (iii) 
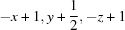
.

**Table 3:** Experimental details

	(I)	(II)
Crystal data		
Chemical formula	C_10_H_13_N_2_^+^·C_8_H_5_Cl_2_O_3_^−^	C_10_H_13_N_2_^+^·C_8_H_5_Cl_2_O_3_^−^
*M*_r_	381.25	381.24
Crystal system, space group	Monoclinic, *P*2_1_	Monoclinic, *P*2_1_
Temperature (K)	200	200
*a*, *b*, *c* (Å)	8.9818 (11), 6.8899 (7), 14.6850 (15)	9.5154 (8), 6.1951 (5), 15.3646 (9)
β (°)	93.565 (9)	102.579 (7)
*V* (Å^3^)	907.00 (17)	883.99 (12)
*Z*	2	2
Radiation type	Mo *K*α	Mo *K*α
μ (mm^−1^)	0.38	0.39
Crystal size (mm)	0.50 × 0.15 × 0.05	0.50 × 0.12 × 0.06

Data collection		
Diffractometer	Oxford Diffraction Gemini-S CCD-detector	Oxford Diffraction Gemini-S CCD-detector
Absorption correction	Multi-scan (*CrysAlis PRO*; Agilent, 2013 ▸)	Multi-scan (*CrysAlis PRO*; Agilent, 2013 ▸)
*T*_min_, *T*_max_	0.940, 0.990	0.872, 0.980
No. of measured, independent and observed [*I* > 2σ(*I*)] reflections	3991, 2896, 2299	3845, 2800, 2451
*R*_int_	0.035	0.027
(sin θ/λ)_max_ (Å^−1^)	0.617	0.617

Refinement		
*R*[*F* ^2^ > 2σ(*F* ^2^)], *wR*(*F* ^2^), *S*	0.068, 0.187, 1.06	0.042, 0.105, 1.08
No. of reflections	2896	2800
No. of parameters	226	238
No. of restraints	1	5
H-atom treatment	H atoms treated by a mixture of independent and constrained refinement	H atoms treated by a mixture of independent and constrained refinement
Δρ_max_, Δρ_min_ (e Å^−3^)	0.37, −0.26	0.22, −0.24
Absolute structure	Flack (1983 ▸)	Flack (1983 ▸)
Absolute structure parameter	0.45 (15)	0.01 (7)

Computer programs: *CrysAlis PRO* (Agilent, 2013 ▸), *SIR92* (Altomare *et al.*, 1993 ▸), *SHELX97* (Sheldrick, 2008 ▸) within *WinGX* (Farrugia, 2012 ▸) and *PLATON* (Spek, 2009 ▸).

## supplementary crystallographic information

### (I) 2-(1H-Indol-3-yl)ethanaminium (2,4-dichlorophenoxy)acetate Crystal data

**Table:**

C_10_H_13_N_2_^+^·C_8_H_5_Cl_2_O_3_^−^	*F*(000) = 396
*M**_r_* = 381.25	*D*_x_ = 1.396 Mg m^−^^3^
Monoclinic, *P*2_1_	Mo *K*α radiation, λ = 0.71073 Å
Hall symbol: P 2yb	Cell parameters from 940 reflections
*a* = 8.9818 (11) Å	θ = 3.8–24.0°
*b* = 6.8899 (7) Å	µ = 0.38 mm^−^^1^
*c* = 14.6850 (15) Å	*T* = 200 K
β = 93.565 (9)°	Needle, colourless
*V* = 907.00 (17) Å^3^	0.50 × 0.15 × 0.05 mm
*Z* = 2	

### (I) 2-(1H-Indol-3-yl)ethanaminium (2,4-dichlorophenoxy)acetate Data collection

**Table:**

Oxford Diffraction Gemini-S CCD-detector diffractometer	2896 independent reflections
Radiation source: Enhance (Mo) X-ray source	2299 reflections with *I* > 2σ(*I*)
Graphite monochromator	*R*_int_ = 0.035
Detector resolution: 16.077 pixels mm^-1^	θ_max_ = 26.0°, θ_min_ = 3.3°
ω scans	*h* = −10→11
Absorption correction: multi-scan (*CrysAlis PRO*; Agilent, 2013)	*k* = −7→8
*T*_min_ = 0.940, *T*_max_ = 0.990	*l* = −17→18
3991 measured reflections	

### (I) 2-(1H-Indol-3-yl)ethanaminium (2,4-dichlorophenoxy)acetate Refinement

**Table:**

Refinement on *F*^2^	Secondary atom site location: difference Fourier map
Least-squares matrix: full	Hydrogen site location: inferred from neighbouring sites
*R*[*F*^2^ > 2σ(*F*^2^)] = 0.068	H atoms treated by a mixture of independent and constrained refinement
*wR*(*F*^2^) = 0.187	*w* = 1/[σ^2^(*F*_o_^2^) + (0.0918*P*)^2^ + 0.3461*P*] where *P* = (*F*_o_^2^ + 2*F*_c_^2^)/3
*S* = 1.06	(Δ/σ)_max_ < 0.001
2896 reflections	Δρ_max_ = 0.37 e Å^−^^3^
226 parameters	Δρ_min_ = −0.26 e Å^−^^3^
1 restraint	Absolute structure: Flack (1983)
Primary atom site location: structure-invariant direct methods	Absolute structure parameter: 0.45 (15)

### (I) 2-(1H-Indol-3-yl)ethanaminium (2,4-dichlorophenoxy)acetate Special details

**Table:**

Geometry. Bond distances, angles etc. have been calculated using the rounded fractional coordinates. All su's are estimated from the variances of the (full) variance-covariance matrix. The cell esds are taken into account in the estimation of distances, angles and torsion angles
Refinement. Refinement of F^2^ against ALL reflections. The weighted R-factor wR and goodness of fit S are based on F^2^, conventional R-factors R are based on F, with F set to zero for negative F^2^. The threshold expression of F^2^ > 2sigma(F^2^) is used only for calculating R-factors(gt) etc. and is not relevant to the choice of reflections for refinement. R-factors based on F^2^ are statistically about twice as large as those based on F, and R- factors based on ALL data will be even larger.

### (I) 2-(1H-Indol-3-yl)ethanaminium (2,4-dichlorophenoxy)acetate Fractional atomic coordinates and isotropic or equivalent isotropic displacement parameters (Å^2^)

**Table:**

	*x*	*y*	*z*	*U*_iso_*/*U*_eq_	
Cl2B	0.28249 (19)	0.2639 (3)	0.84021 (11)	0.0625 (6)	
Cl4B	0.8117 (2)	0.2352 (4)	1.03185 (12)	0.0817 (8)	
O11B	0.3858 (5)	0.6285 (7)	0.7750 (3)	0.0565 (16)	
O13B	0.5619 (4)	0.6213 (5)	0.6335 (3)	0.0378 (12)	
O14B	0.5207 (4)	0.9327 (5)	0.6033 (3)	0.0346 (11)	
C1B	0.4889 (7)	0.5483 (10)	0.8340 (4)	0.0444 (19)	
C2B	0.4569 (7)	0.3677 (10)	0.8717 (4)	0.047 (2)	
C3B	0.5495 (7)	0.2673 (11)	0.9322 (3)	0.049 (2)	
C4B	0.6859 (8)	0.3564 (12)	0.9564 (4)	0.057 (3)	
C5B	0.7253 (7)	0.5320 (11)	0.9216 (4)	0.051 (2)	
C6B	0.6281 (8)	0.6256 (11)	0.8585 (4)	0.055 (2)	
C12B	0.4160 (7)	0.8064 (9)	0.7333 (4)	0.045 (2)	
C13B	0.5129 (5)	0.7839 (8)	0.6498 (3)	0.0290 (17)	
N1A	0.7673 (5)	1.3071 (7)	0.6100 (3)	0.0397 (16)	
N32A	0.6027 (5)	0.7972 (7)	0.4326 (3)	0.0317 (14)	
C2A	0.7670 (5)	1.2445 (7)	0.5215 (3)	0.0262 (14)	
C3A	0.8329 (5)	1.0707 (7)	0.5166 (3)	0.0274 (16)	
C4A	0.9521 (6)	0.8575 (9)	0.6506 (4)	0.0410 (17)	
C5A	0.9790 (7)	0.8583 (11)	0.7434 (4)	0.052 (2)	
C6A	0.9345 (8)	1.0100 (12)	0.7973 (5)	0.060 (3)	
C7A	0.8638 (7)	1.1725 (11)	0.7595 (4)	0.055 (2)	
C8A	0.8359 (6)	1.1754 (9)	0.6647 (4)	0.0388 (19)	
C9A	0.8793 (5)	1.0155 (8)	0.6100 (4)	0.0296 (16)	
C31A	0.8527 (6)	0.9501 (7)	0.4333 (3)	0.0304 (17)	
C32A	0.7656 (5)	0.7611 (8)	0.4290 (3)	0.0296 (16)	
H3B	0.52290	0.14500	0.95620	0.0580*	
H5B	0.81840	0.58950	0.94030	0.0620*	
H6B	0.65760	0.74420	0.83190	0.0660*	
H12B	0.46840	0.89250	0.77880	0.0550*	
H13B	0.32040	0.86940	0.71320	0.0550*	
H1A	0.743 (7)	1.423 (5)	0.627 (4)	0.0480*	
H2A	0.72550	1.31490	0.47040	0.0310*	
H4A	0.98260	0.75130	0.61500	0.0490*	
H5A	1.02980	0.75130	0.77180	0.0620*	
H6A	0.95280	1.00250	0.86160	0.0720*	
H7A	0.83540	1.27770	0.79650	0.0660*	
H30B	0.96010	0.91970	0.43050	0.0360*	
H31B	0.82230	1.02830	0.37880	0.0360*	
H32B	0.78460	0.69190	0.37180	0.0360*	
H33B	0.80010	0.67720	0.48090	0.0360*	
H34A	0.559 (6)	0.684 (5)	0.419 (3)	0.0380*	
H35A	0.581 (6)	0.883 (6)	0.388 (3)	0.0380*	
H36A	0.581 (6)	0.828 (8)	0.4889 (19)	0.0380*	

### (I) 2-(1H-Indol-3-yl)ethanaminium (2,4-dichlorophenoxy)acetate Atomic displacement parameters (Å^2^)

**Table:**

	*U*^11^	*U*^22^	*U*^33^	*U*^12^	*U*^13^	*U*^23^
Cl2B	0.0639 (11)	0.0664 (11)	0.0572 (10)	−0.0152 (10)	0.0030 (7)	−0.0014 (9)
Cl4B	0.0779 (13)	0.1142 (19)	0.0512 (10)	0.0072 (13)	−0.0110 (8)	0.0160 (11)
O11B	0.055 (3)	0.064 (3)	0.051 (2)	0.004 (2)	0.007 (2)	0.017 (2)
O13B	0.044 (2)	0.024 (2)	0.045 (2)	0.0037 (17)	−0.0007 (17)	0.0043 (16)
O14B	0.0250 (19)	0.029 (2)	0.050 (2)	−0.0007 (16)	0.0030 (15)	0.0017 (17)
C1B	0.047 (3)	0.055 (4)	0.032 (3)	0.005 (3)	0.009 (2)	−0.002 (3)
C2B	0.063 (4)	0.050 (4)	0.029 (3)	0.004 (3)	0.009 (3)	−0.005 (3)
C3B	0.069 (4)	0.049 (4)	0.029 (3)	−0.005 (3)	0.011 (2)	0.004 (3)
C4B	0.069 (5)	0.074 (5)	0.028 (3)	0.007 (4)	0.012 (3)	0.005 (3)
C5B	0.036 (3)	0.064 (5)	0.054 (4)	−0.010 (3)	0.004 (3)	−0.008 (4)
C6B	0.059 (4)	0.059 (4)	0.047 (4)	0.000 (4)	0.008 (3)	0.004 (3)
C12B	0.048 (4)	0.042 (4)	0.047 (3)	0.016 (3)	0.010 (3)	0.015 (3)
C13B	0.028 (3)	0.025 (3)	0.033 (3)	0.006 (2)	−0.0060 (18)	−0.002 (2)
N1A	0.031 (2)	0.028 (3)	0.060 (3)	0.004 (2)	0.003 (2)	−0.009 (2)
N32A	0.024 (2)	0.035 (3)	0.036 (2)	−0.003 (2)	0.0010 (17)	0.000 (2)
C2A	0.019 (2)	0.016 (2)	0.043 (3)	−0.004 (2)	−0.0016 (18)	0.001 (2)
C3A	0.018 (2)	0.023 (3)	0.041 (3)	0.000 (2)	0.0008 (19)	−0.004 (2)
C4A	0.030 (3)	0.043 (3)	0.050 (3)	0.013 (3)	0.003 (2)	0.003 (3)
C5A	0.042 (3)	0.062 (4)	0.050 (4)	0.013 (3)	−0.008 (3)	0.000 (3)
C6A	0.068 (5)	0.072 (5)	0.041 (4)	−0.016 (4)	0.003 (3)	−0.002 (4)
C7A	0.045 (4)	0.075 (5)	0.045 (3)	0.003 (4)	0.009 (3)	−0.012 (3)
C8A	0.027 (3)	0.044 (4)	0.046 (3)	−0.002 (3)	0.008 (2)	−0.005 (3)
C9A	0.020 (2)	0.027 (3)	0.042 (3)	−0.005 (2)	0.0047 (19)	−0.003 (2)
C31A	0.026 (3)	0.030 (3)	0.035 (3)	−0.002 (2)	0.001 (2)	−0.004 (2)
C32A	0.025 (2)	0.023 (3)	0.041 (3)	−0.001 (2)	0.0029 (19)	−0.001 (2)

### (I) 2-(1H-Indol-3-yl)ethanaminium (2,4-dichlorophenoxy)acetate Geometric parameters (Å, º)

**Table:**

Cl2B—C2B	1.758 (7)	C6B—H6B	0.9500
Cl4B—C4B	1.745 (7)	C12B—H13B	0.9900
O11B—C1B	1.347 (8)	C12B—H12B	0.9900
O11B—C12B	1.404 (8)	C2A—C3A	1.340 (7)
O13B—C13B	1.233 (6)	C3A—C9A	1.459 (7)
O14B—C13B	1.236 (6)	C3A—C31A	1.499 (6)
N1A—C8A	1.337 (8)	C4A—C5A	1.369 (8)
N1A—C2A	1.369 (6)	C4A—C9A	1.386 (8)
N32A—C32A	1.488 (6)	C5A—C6A	1.385 (10)
N1A—H1A	0.87 (4)	C6A—C7A	1.386 (11)
N32A—H34A	0.89 (4)	C7A—C8A	1.399 (8)
N32A—H36A	0.89 (3)	C8A—C9A	1.432 (8)
N32A—H35A	0.90 (4)	C31A—C32A	1.518 (7)
C1B—C2B	1.399 (9)	C2A—H2A	0.9500
C1B—C6B	1.386 (10)	C4A—H4A	0.9500
C2B—C3B	1.367 (9)	C5A—H5A	0.9500
C3B—C4B	1.396 (10)	C6A—H6A	0.9500
C4B—C5B	1.369 (11)	C7A—H7A	0.9500
C5B—C6B	1.391 (9)	C31A—H30B	0.9900
C12B—C13B	1.555 (7)	C31A—H31B	0.9900
C3B—H3B	0.9500	C32A—H32B	0.9900
C5B—H5B	0.9500	C32A—H33B	0.9900
			
C1B—O11B—C12B	119.7 (5)	N1A—C2A—C3A	111.0 (4)
C2A—N1A—C8A	109.3 (5)	C2A—C3A—C9A	106.5 (4)
C8A—N1A—H1A	125 (4)	C2A—C3A—C31A	127.9 (4)
C2A—N1A—H1A	125 (4)	C9A—C3A—C31A	125.6 (4)
C32A—N32A—H35A	105 (3)	C5A—C4A—C9A	118.3 (6)
H34A—N32A—H36A	107 (5)	C4A—C5A—C6A	122.2 (7)
C32A—N32A—H36A	110 (3)	C5A—C6A—C7A	121.5 (6)
H34A—N32A—H35A	110 (4)	C6A—C7A—C8A	117.3 (6)
C32A—N32A—H34A	105 (3)	N1A—C8A—C9A	108.5 (5)
H35A—N32A—H36A	118 (4)	C7A—C8A—C9A	120.6 (6)
O11B—C1B—C2B	118.0 (6)	N1A—C8A—C7A	130.9 (6)
C2B—C1B—C6B	116.3 (6)	C3A—C9A—C4A	135.2 (5)
O11B—C1B—C6B	125.6 (6)	C3A—C9A—C8A	104.8 (5)
Cl2B—C2B—C1B	117.4 (5)	C4A—C9A—C8A	120.1 (5)
C1B—C2B—C3B	125.2 (6)	C3A—C31A—C32A	115.0 (4)
Cl2B—C2B—C3B	117.5 (5)	N32A—C32A—C31A	111.1 (4)
C2B—C3B—C4B	115.6 (6)	N1A—C2A—H2A	125.00
Cl4B—C4B—C5B	119.2 (5)	C3A—C2A—H2A	125.00
Cl4B—C4B—C3B	118.3 (6)	C5A—C4A—H4A	121.00
C3B—C4B—C5B	122.5 (6)	C9A—C4A—H4A	121.00
C4B—C5B—C6B	119.5 (6)	C4A—C5A—H5A	119.00
C1B—C6B—C5B	120.9 (7)	C6A—C5A—H5A	119.00
O11B—C12B—C13B	112.9 (5)	C5A—C6A—H6A	119.00
O13B—C13B—O14B	127.8 (5)	C7A—C6A—H6A	119.00
O13B—C13B—C12B	117.9 (5)	C6A—C7A—H7A	121.00
O14B—C13B—C12B	114.0 (5)	C8A—C7A—H7A	121.00
C2B—C3B—H3B	122.00	C3A—C31A—H30B	108.00
C4B—C3B—H3B	122.00	C3A—C31A—H31B	109.00
C6B—C5B—H5B	120.00	C32A—C31A—H30B	109.00
C4B—C5B—H5B	120.00	C32A—C31A—H31B	109.00
C1B—C6B—H6B	120.00	H30B—C31A—H31B	108.00
C5B—C6B—H6B	120.00	N32A—C32A—H32B	109.00
O11B—C12B—H13B	109.00	N32A—C32A—H33B	109.00
C13B—C12B—H13B	109.00	C31A—C32A—H32B	109.00
H12B—C12B—H13B	108.00	C31A—C32A—H33B	109.00
C13B—C12B—H12B	109.00	H32B—C32A—H33B	108.00
O11B—C12B—H12B	109.00		
			
C12B—O11B—C1B—C2B	−177.8 (5)	N1A—C2A—C3A—C9A	0.3 (5)
C12B—O11B—C1B—C6B	−0.5 (9)	N1A—C2A—C3A—C31A	178.5 (5)
C1B—O11B—C12B—C13B	81.2 (6)	C2A—C3A—C9A—C4A	−179.6 (6)
C8A—N1A—C2A—C3A	0.9 (6)	C2A—C3A—C9A—C8A	−1.3 (5)
C2A—N1A—C8A—C7A	−179.8 (6)	C31A—C3A—C9A—C4A	2.1 (9)
C2A—N1A—C8A—C9A	−1.7 (6)	C31A—C3A—C9A—C8A	−179.6 (5)
C6B—C1B—C2B—C3B	2.3 (10)	C2A—C3A—C31A—C32A	−113.1 (5)
O11B—C1B—C6B—C5B	178.8 (6)	C9A—C3A—C31A—C32A	64.8 (6)
C2B—C1B—C6B—C5B	−3.8 (9)	C9A—C4A—C5A—C6A	0.7 (9)
O11B—C1B—C2B—C3B	179.9 (6)	C5A—C4A—C9A—C3A	179.0 (6)
C6B—C1B—C2B—Cl2B	−178.9 (5)	C5A—C4A—C9A—C8A	0.8 (8)
O11B—C1B—C2B—Cl2B	−1.3 (8)	C4A—C5A—C6A—C7A	−1.9 (11)
Cl2B—C2B—C3B—C4B	−179.1 (5)	C5A—C6A—C7A—C8A	1.5 (10)
C1B—C2B—C3B—C4B	−0.2 (9)	C6A—C7A—C8A—N1A	178.0 (6)
C2B—C3B—C4B—Cl4B	−178.9 (5)	C6A—C7A—C8A—C9A	0.1 (9)
C2B—C3B—C4B—C5B	−0.4 (9)	N1A—C8A—C9A—C3A	1.8 (6)
C3B—C4B—C5B—C6B	−1.1 (10)	N1A—C8A—C9A—C4A	−179.5 (5)
Cl4B—C4B—C5B—C6B	177.3 (5)	C7A—C8A—C9A—C3A	−179.9 (5)
C4B—C5B—C6B—C1B	3.4 (10)	C7A—C8A—C9A—C4A	−1.2 (8)
O11B—C12B—C13B—O13B	−5.6 (7)	C3A—C31A—C32A—N32A	58.6 (5)
O11B—C12B—C13B—O14B	168.9 (5)		

### (I) 2-(1H-Indol-3-yl)ethanaminium (2,4-dichlorophenoxy)acetate Hydrogen-bond geometry (Å, º)

**Table:**

*D*—H···*A*	*D*—H	H···*A*	*D*···*A*	*D*—H···*A*
N1*A*—H1*A*···O13*B*^i^	0.87 (4)	2.13 (5)	2.879 (6)	144 (6)
N32*A*—H34*A*···O14*B*^ii^	0.89 (4)	1.89 (4)	2.782 (6)	175 (2)
N32*A*—H35*A*···O13*B*^iii^	0.90 (4)	2.10 (5)	2.817 (6)	137 (4)
N32*A*—H36*A*···O13*B*	0.89 (3)	2.57 (4)	3.231 (6)	132 (4)
N32*A*—H36*A*···O14*B*	0.89 (3)	1.94 (4)	2.816 (6)	171 (5)
C2*A*—H2*A*···O14*B*^iii^	0.95	2.53	3.336 (6)	142

Symmetry codes: (i) *x*, *y*+1, *z*; (ii) −*x*+1, *y*−1/2, −*z*+1; (iii) −*x*+1, *y*+1/2, −*z*+1.

### (II) 2-(1H-Indol-3-yl)ethanaminium (3,5-dichlorophenoxy)acetate Crystal data

**Table:**

C_10_H_13_N_2_^+^·C_8_H_5_Cl_2_O_3_^−^	*F*(000) = 396
*M**_r_* = 381.24	*D*_x_ = 1.432 Mg m^−^^3^
Monoclinic, *P*2_1_	Mo *K*α radiation, λ = 0.71073 Å
Hall symbol: P 2yb	Cell parameters from 1140 reflections
*a* = 9.5154 (8) Å	θ = 3.9–28.2°
*b* = 6.1951 (5) Å	µ = 0.39 mm^−^^1^
*c* = 15.3646 (9) Å	*T* = 200 K
β = 102.579 (7)°	Needle, colourless
*V* = 883.99 (12) Å^3^	0.50 × 0.12 × 0.06 mm
*Z* = 2	

### (II) 2-(1H-Indol-3-yl)ethanaminium (3,5-dichlorophenoxy)acetate Data collection

**Table:**

Oxford Diffraction Gemini-S CCD-detector diffractometer	2800 independent reflections
Radiation source: fine-focus sealed tube	2451 reflections with *I* > 2σ(*I*)
Graphite monochromator	*R*_int_ = 0.027
Detector resolution: 16.077 pixels mm^-1^	θ_max_ = 26.0°, θ_min_ = 3.1°
ω scans	*h* = −11→7
Absorption correction: multi-scan (*CrysAlis PRO*; Agilent, 2013)	*k* = −7→7
*T*_min_ = 0.872, *T*_max_ = 0.980	*l* = −18→18
3845 measured reflections	

### (II) 2-(1H-Indol-3-yl)ethanaminium (3,5-dichlorophenoxy)acetate Refinement

**Table:**

Refinement on *F*^2^	Secondary atom site location: difference Fourier map
Least-squares matrix: full	Hydrogen site location: inferred from neighbouring sites
*R*[*F*^2^ > 2σ(*F*^2^)] = 0.042	H atoms treated by a mixture of independent and constrained refinement
*wR*(*F*^2^) = 0.105	*w* = 1/[σ^2^(*F*_o_^2^) + (0.0513*P*)^2^] where *P* = (*F*_o_^2^ + 2*F*_c_^2^)/3
*S* = 1.08	(Δ/σ)_max_ = 0.001
2800 reflections	Δρ_max_ = 0.22 e Å^−^^3^
238 parameters	Δρ_min_ = −0.24 e Å^−^^3^
5 restraints	Absolute structure: Flack (1983)
Primary atom site location: structure-invariant direct methods	Absolute structure parameter: 0.01 (7)

### (II) 2-(1H-Indol-3-yl)ethanaminium (3,5-dichlorophenoxy)acetate Special details

**Table:**

Geometry. Bond distances, angles etc. have been calculated using the rounded fractional coordinates. All su's are estimated from the variances of the (full) variance-covariance matrix. The cell esds are taken into account in the estimation of distances, angles and torsion angles
Refinement. Refinement of F^2^ against ALL reflections. The weighted R-factor wR and goodness of fit S are based on F^2^, conventional R-factors R are based on F, with F set to zero for negative F^2^. The threshold expression of F^2^ > 2sigma(F^2^) is used only for calculating R-factors(gt) etc. and is not relevant to the choice of reflections for refinement. R-factors based on F^2^ are statistically about twice as large as those based on F, and R- factors based on ALL data will be even larger.

### (II) 2-(1H-Indol-3-yl)ethanaminium (3,5-dichlorophenoxy)acetate Fractional atomic coordinates and isotropic or equivalent isotropic displacement parameters (Å^2^)

**Table:**

	*x*	*y*	*z*	*U*_iso_*/*U*_eq_	
Cl3B	0.88773 (13)	1.0762 (2)	0.94414 (6)	0.0556 (4)	
Cl5B	0.61601 (10)	0.46571 (17)	1.09183 (5)	0.0421 (3)	
O11B	0.6022 (3)	0.4935 (4)	0.75745 (13)	0.0337 (8)	
O13B	0.5666 (3)	0.2827 (4)	0.60040 (14)	0.0269 (7)	
O14B	0.4608 (3)	0.0059 (4)	0.65216 (15)	0.0353 (8)	
C1B	0.6435 (3)	0.5773 (6)	0.84115 (19)	0.0266 (10)	
C2B	0.7290 (4)	0.7600 (6)	0.8496 (2)	0.0304 (10)	
C3B	0.7776 (4)	0.8496 (6)	0.9324 (2)	0.0301 (11)	
C4B	0.7436 (4)	0.7632 (6)	1.0088 (2)	0.0306 (10)	
C5B	0.6601 (4)	0.5807 (7)	0.99749 (19)	0.0298 (10)	
C6B	0.6062 (3)	0.4868 (6)	0.91596 (19)	0.0279 (10)	
C12B	0.5268 (4)	0.2927 (6)	0.7495 (2)	0.0297 (11)	
C13B	0.5178 (3)	0.1909 (5)	0.6593 (2)	0.0243 (10)	
N1A	0.2443 (3)	0.6837 (5)	0.6332 (2)	0.0339 (10)	
N32A	0.3860 (3)	0.2111 (5)	0.42680 (18)	0.0238 (8)	
C2A	0.2536 (3)	0.5745 (7)	0.5567 (2)	0.0314 (11)	
C3A	0.1828 (3)	0.3819 (6)	0.5518 (2)	0.0238 (10)	
C4A	0.0375 (4)	0.2219 (7)	0.6612 (2)	0.0366 (11)	
C5A	−0.0092 (4)	0.2656 (7)	0.7385 (2)	0.0435 (14)	
C6A	0.0307 (4)	0.4575 (8)	0.7856 (2)	0.0420 (13)	
C7A	0.1174 (4)	0.6077 (7)	0.7582 (2)	0.0366 (11)	
C8A	0.1640 (3)	0.5635 (6)	0.6792 (2)	0.0293 (10)	
C9A	0.1237 (3)	0.3729 (6)	0.6306 (2)	0.0258 (10)	
C31A	0.1548 (4)	0.2190 (6)	0.4786 (2)	0.0307 (11)	
C32A	0.2298 (3)	0.2630 (6)	0.4028 (2)	0.0290 (10)	
H2B	0.75380	0.82280	0.79850	0.0360*	
H4B	0.77660	0.82720	1.06580	0.0370*	
H6B	0.54530	0.36380	0.91080	0.0340*	
H12B	0.42830	0.31680	0.75890	0.0350*	
H13B	0.57670	0.19270	0.79650	0.0350*	
H1A	0.291 (4)	0.803 (5)	0.649 (3)	0.0530*	
H2A	0.30260	0.62610	0.51320	0.0380*	
H4A	0.01130	0.09110	0.62960	0.0440*	
H5A	−0.06890	0.16460	0.75980	0.0530*	
H6A	−0.00350	0.48420	0.83830	0.0500*	
H7A	0.14490	0.73640	0.79120	0.0440*	
H30A	0.04970	0.21190	0.45400	0.0370*	
H31A	0.18580	0.07560	0.50430	0.0370*	
H32A	0.18350	0.17590	0.35030	0.0350*	
H33A	0.21760	0.41720	0.38590	0.0350*	
H34A	0.420 (4)	0.237 (7)	0.4830 (14)	0.0530*	
H35A	0.407 (4)	0.080 (4)	0.409 (3)	0.0530*	
H36A	0.438 (4)	0.292 (7)	0.398 (3)	0.0530*	

### (II) 2-(1H-Indol-3-yl)ethanaminium (3,5-dichlorophenoxy)acetate Atomic displacement parameters (Å^2^)

**Table:**

	*U*^11^	*U*^22^	*U*^33^	*U*^12^	*U*^13^	*U*^23^
Cl3B	0.0674 (7)	0.0572 (7)	0.0408 (5)	−0.0354 (6)	0.0090 (5)	−0.0097 (5)
Cl5B	0.0558 (6)	0.0482 (6)	0.0235 (4)	−0.0057 (5)	0.0112 (4)	0.0014 (4)
O11B	0.0576 (15)	0.0254 (14)	0.0187 (10)	−0.0111 (13)	0.0099 (10)	−0.0035 (10)
O13B	0.0352 (13)	0.0241 (13)	0.0215 (11)	−0.0037 (11)	0.0061 (10)	−0.0029 (10)
O14B	0.0435 (14)	0.0235 (15)	0.0423 (13)	−0.0097 (12)	0.0168 (11)	−0.0078 (11)
C1B	0.0358 (18)	0.0236 (18)	0.0205 (15)	−0.0006 (16)	0.0066 (14)	−0.0050 (15)
C2B	0.0355 (18)	0.032 (2)	0.0243 (16)	−0.0005 (17)	0.0081 (15)	0.0001 (16)
C3B	0.0319 (19)	0.027 (2)	0.0300 (17)	−0.0034 (16)	0.0038 (16)	−0.0043 (16)
C4B	0.0289 (17)	0.037 (2)	0.0240 (16)	−0.0029 (16)	0.0015 (14)	−0.0103 (16)
C5B	0.0341 (18)	0.034 (2)	0.0215 (15)	0.0066 (18)	0.0066 (13)	0.0039 (16)
C6B	0.0336 (17)	0.0249 (19)	0.0251 (15)	−0.0020 (16)	0.0059 (14)	0.0008 (15)
C12B	0.042 (2)	0.024 (2)	0.0236 (16)	−0.0046 (17)	0.0085 (15)	−0.0044 (15)
C13B	0.0266 (17)	0.0200 (19)	0.0257 (15)	−0.0023 (16)	0.0041 (14)	0.0012 (15)
N1A	0.0349 (17)	0.0248 (17)	0.0427 (16)	−0.0066 (14)	0.0099 (14)	−0.0069 (15)
N32A	0.0264 (14)	0.0230 (17)	0.0216 (12)	0.0016 (13)	0.0044 (12)	0.0005 (13)
C2A	0.0261 (17)	0.033 (2)	0.0366 (18)	−0.0040 (17)	0.0103 (14)	−0.0023 (17)
C3A	0.0202 (16)	0.0249 (19)	0.0268 (16)	−0.0021 (14)	0.0064 (14)	0.0008 (15)
C4A	0.041 (2)	0.034 (2)	0.0362 (18)	−0.0090 (19)	0.0115 (17)	0.0043 (17)
C5A	0.044 (2)	0.052 (3)	0.038 (2)	−0.008 (2)	0.0163 (19)	0.007 (2)
C6A	0.040 (2)	0.061 (3)	0.0260 (16)	0.009 (2)	0.0092 (16)	0.004 (2)
C7A	0.0336 (19)	0.044 (2)	0.0280 (17)	0.0066 (18)	−0.0026 (15)	−0.0081 (18)
C8A	0.0212 (16)	0.035 (2)	0.0297 (17)	0.0061 (16)	0.0010 (14)	0.0006 (17)
C9A	0.0213 (16)	0.028 (2)	0.0262 (16)	0.0012 (15)	0.0012 (14)	0.0018 (15)
C31A	0.0281 (17)	0.029 (2)	0.0348 (18)	−0.0045 (16)	0.0062 (15)	−0.0042 (16)
C32A	0.0267 (17)	0.032 (2)	0.0269 (17)	0.0044 (16)	0.0027 (15)	−0.0013 (16)

### (II) 2-(1H-Indol-3-yl)ethanaminium (3,5-dichlorophenoxy)acetate Geometric parameters (Å, º)

**Table:**

Cl3B—C3B	1.738 (4)	C6B—H6B	0.9500
Cl5B—C5B	1.746 (3)	C12B—H13B	0.9900
O11B—C1B	1.363 (4)	C12B—H12B	0.9900
O11B—C12B	1.428 (5)	C2A—C3A	1.364 (5)
O13B—C13B	1.241 (4)	C3A—C9A	1.443 (4)
O14B—C13B	1.263 (4)	C3A—C31A	1.491 (5)
N1A—C8A	1.369 (4)	C4A—C5A	1.383 (5)
N1A—C2A	1.376 (5)	C4A—C9A	1.392 (5)
N32A—C32A	1.487 (4)	C5A—C6A	1.401 (6)
N1A—H1A	0.87 (4)	C6A—C7A	1.369 (6)
N32A—H34A	0.87 (2)	C7A—C8A	1.407 (4)
N32A—H36A	0.89 (4)	C8A—C9A	1.405 (5)
N32A—H35A	0.89 (3)	C31A—C32A	1.517 (5)
C1B—C2B	1.383 (5)	C2A—H2A	0.9500
C1B—C6B	1.393 (4)	C4A—H4A	0.9500
C2B—C3B	1.373 (4)	C5A—H5A	0.9500
C3B—C4B	1.391 (5)	C6A—H6A	0.9500
C4B—C5B	1.371 (6)	C7A—H7A	0.9500
C5B—C6B	1.375 (4)	C31A—H30A	0.9900
C12B—C13B	1.508 (4)	C31A—H31A	0.9900
C2B—H2B	0.9500	C32A—H32A	0.9900
C4B—H4B	0.9500	C32A—H33A	0.9900
			
C1B—O11B—C12B	116.7 (2)	N1A—C2A—C3A	110.8 (3)
C2A—N1A—C8A	108.7 (3)	C2A—C3A—C9A	105.4 (3)
C8A—N1A—H1A	129 (3)	C2A—C3A—C31A	129.7 (3)
C2A—N1A—H1A	122 (3)	C9A—C3A—C31A	124.6 (3)
C32A—N32A—H35A	114 (3)	C5A—C4A—C9A	118.8 (4)
H34A—N32A—H36A	105 (4)	C4A—C5A—C6A	120.6 (4)
C32A—N32A—H36A	113 (3)	C5A—C6A—C7A	122.1 (3)
H34A—N32A—H35A	114 (4)	C6A—C7A—C8A	117.2 (4)
C32A—N32A—H34A	110 (3)	N1A—C8A—C9A	107.5 (3)
H35A—N32A—H36A	100 (4)	C7A—C8A—C9A	121.4 (3)
O11B—C1B—C2B	116.3 (3)	N1A—C8A—C7A	131.0 (3)
C2B—C1B—C6B	120.2 (3)	C3A—C9A—C4A	132.5 (3)
O11B—C1B—C6B	123.5 (3)	C3A—C9A—C8A	107.6 (3)
C1B—C2B—C3B	119.4 (3)	C4A—C9A—C8A	119.9 (3)
C2B—C3B—C4B	122.2 (3)	C3A—C31A—C32A	114.9 (3)
Cl3B—C3B—C4B	118.0 (3)	N32A—C32A—C31A	112.5 (3)
Cl3B—C3B—C2B	119.8 (3)	N1A—C2A—H2A	125.00
C3B—C4B—C5B	116.4 (3)	C3A—C2A—H2A	125.00
Cl5B—C5B—C4B	117.9 (2)	C5A—C4A—H4A	121.00
Cl5B—C5B—C6B	118.3 (3)	C9A—C4A—H4A	121.00
C4B—C5B—C6B	123.7 (3)	C4A—C5A—H5A	120.00
C1B—C6B—C5B	118.0 (3)	C6A—C5A—H5A	120.00
O11B—C12B—C13B	111.7 (3)	C5A—C6A—H6A	119.00
O13B—C13B—O14B	125.1 (3)	C7A—C6A—H6A	119.00
O13B—C13B—C12B	121.5 (3)	C6A—C7A—H7A	121.00
O14B—C13B—C12B	113.4 (3)	C8A—C7A—H7A	121.00
C1B—C2B—H2B	120.00	C3A—C31A—H30A	109.00
C3B—C2B—H2B	120.00	C3A—C31A—H31A	109.00
C5B—C4B—H4B	122.00	C32A—C31A—H30A	109.00
C3B—C4B—H4B	122.00	C32A—C31A—H31A	109.00
C1B—C6B—H6B	121.00	H30A—C31A—H31A	107.00
C5B—C6B—H6B	121.00	N32A—C32A—H32A	109.00
O11B—C12B—H13B	109.00	N32A—C32A—H33A	109.00
C13B—C12B—H13B	109.00	C31A—C32A—H32A	109.00
H12B—C12B—H13B	108.00	C31A—C32A—H33A	109.00
C13B—C12B—H12B	109.00	H32A—C32A—H33A	108.00
O11B—C12B—H12B	109.00		
			
C12B—O11B—C1B—C2B	173.6 (3)	N1A—C2A—C3A—C9A	0.8 (4)
C12B—O11B—C1B—C6B	−5.2 (5)	N1A—C2A—C3A—C31A	174.8 (3)
C1B—O11B—C12B—C13B	−166.5 (3)	C2A—C3A—C9A—C4A	177.5 (4)
C2A—N1A—C8A—C7A	−176.4 (3)	C2A—C3A—C9A—C8A	−0.3 (4)
C2A—N1A—C8A—C9A	0.8 (4)	C31A—C3A—C9A—C4A	3.1 (6)
C8A—N1A—C2A—C3A	−1.0 (4)	C31A—C3A—C9A—C8A	−174.8 (3)
O11B—C1B—C6B—C5B	177.0 (3)	C2A—C3A—C31A—C32A	7.3 (5)
C2B—C1B—C6B—C5B	−1.8 (5)	C9A—C3A—C31A—C32A	−179.7 (3)
O11B—C1B—C2B—C3B	−178.3 (3)	C9A—C4A—C5A—C6A	−0.6 (6)
C6B—C1B—C2B—C3B	0.6 (5)	C5A—C4A—C9A—C3A	−176.3 (3)
C1B—C2B—C3B—Cl3B	178.8 (3)	C5A—C4A—C9A—C8A	1.3 (5)
C1B—C2B—C3B—C4B	−0.1 (6)	C4A—C5A—C6A—C7A	−0.5 (6)
Cl3B—C3B—C4B—C5B	−178.1 (3)	C5A—C6A—C7A—C8A	0.9 (6)
C2B—C3B—C4B—C5B	0.9 (6)	C6A—C7A—C8A—N1A	176.6 (4)
C3B—C4B—C5B—C6B	−2.3 (6)	C6A—C7A—C8A—C9A	−0.2 (5)
C3B—C4B—C5B—Cl5B	179.5 (3)	N1A—C8A—C9A—C3A	−0.3 (4)
Cl5B—C5B—C6B—C1B	−179.1 (3)	N1A—C8A—C9A—C4A	−178.4 (3)
C4B—C5B—C6B—C1B	2.7 (6)	C7A—C8A—C9A—C3A	177.2 (3)
O11B—C12B—C13B—O13B	−2.9 (5)	C7A—C8A—C9A—C4A	−0.9 (5)
O11B—C12B—C13B—O14B	175.3 (3)	C3A—C31A—C32A—N32A	75.7 (4)

### (II) 2-(1H-Indol-3-yl)ethanaminium (3,5-dichlorophenoxy)acetate Hydrogen-bond geometry (Å, º)

**Table:**

*D*—H···*A*	*D*—H	H···*A*	*D*···*A*	*D*—H···*A*
N1*A*—H1*A*···O14*B*^i^	0.87 (4)	2.04 (4)	2.838 (4)	152 (4)
N32*A*—H34*A*···O13*B*	0.87 (2)	2.05 (3)	2.875 (4)	160 (4)
N32*A*—H35*A*···O11*B*^ii^	0.89 (3)	2.60 (4)	3.160 (4)	122 (3)
N32*A*—H35*A*···O13*B*^ii^	0.89 (3)	1.87 (3)	2.739 (4)	164 (4)
N32*A*—H36*A*···O14*B*^iii^	0.89 (4)	1.90 (4)	2.775 (4)	170 (4)
C2*A*—H2*A*···O13*B*^iii^	0.95	2.55	3.495 (4)	177

Symmetry codes: (i) *x*, *y*+1, *z*; (ii) −*x*+1, *y*−1/2, −*z*+1; (iii) −*x*+1, *y*+1/2, −*z*+1.
